# Tinkering with Blood: Optimizing the Coagulation System for Therapeutic Purposes

**DOI:** 10.3390/bioengineering12121301

**Published:** 2025-11-26

**Authors:** Eduardo Anitua, Sabino Padilla

**Affiliations:** 1 Eduardo Anitua Foundation for Biomedical Research, 01007 Vitoria, Spain; 2 Regenerative Medicine Laboratory, BTI-Biotechnology Institute I MAS D, 01007 Vitoria, Spain; 3 University Institute for Regenerative Medicine & Oral Implantology-UIRMI (UPV/EHU-Fundación Eduardo Anitua), 01007 Vitoria, Spain

**Keywords:** regenerative medicine, blood, platelet-rich plasma, coagulation, growth factors, thromboinflammation

## Abstract

Blood is a multitask, fluid tissue that is considered as an endless goldmine for regenerative therapies. This connective tissue carries myriad multidomain proteins as the workhorse of biological functions integrated in complex molecular networks. Among them, the coagulation system stands out, with platelets and plasma coagulation proteins playing multiple roles in clotting, defense and tissue repair, the latter of which is the final byproduct process stemming from the hemostatic–inflammatory, cell-reprogramming and inflammation resolution after a tissue injury. By mimicking coagulation and hemostasis but lacking inflammatory properties, platelet-rich plasma (PRP) is emerging as an innovative autologous therapy operating as a local delivery system of growth factors. Processing of the patient blood to manufacture PRP encompasses blood anticoagulation; blood deconstruction through centrifugation and fractionation; and activation of plasma, endowing the applied product with anti-inflammatory, trophic, antifibrotic and antialgic properties in a context-dependent manner. However, the field of PRPs faces controversies due to the heterogeneity of their biological compositions and modalities of application. Moreover, there are some drawbacks derived from patient age and some other conditions, all impinging negatively on PRP clinical outcomes. Standardization of the manufacturing process, elaboration of guidelines of application and use of allogenic PRPs are emerging as possible solutions to surmount these pitfalls.

## 1. Introduction

The early 21st century is witnessing an unprecedented interest in imitating models from nature (biomimetics) to design material in the field of tissue engineering, with biology serving as a source of inspiration for problem solving in areas ranging from architecture and robotics to engineering, biology and medicine [1,2]. But a biomimetic approach to innovation is not new in the field of medicine. One biological source is blood, which is not only a window through which scientists seek to unravel disease, regeneration and aging [3,4,5] but also an endless goldmine for regenerative therapies [6,7,8]. More than 80 years ago (1942), heralding the field of biomimetics and biomedicine, two British surgeons, H.J. Seddon and P.B. Medawar (1942), described the first procedure for suturing peripheral nerves in humans with fibrin obtained from rooster plasma, achieving satisfactory results [9]. Nevertheless, was not until the beginning of the 21st century when autologous blood-derived products, popularized under the generic term of platelet-rich plasmas (PRPs), began to show exponential growth both in research and in their clinical applications, as reflected in the bibliometrics of scientific publications [10,11]. Although platelet-rich plasma (PRP) and platelet-poor plasma (PPP) have long clinical histories as samples of coagulation assays to assess ex vivo plasma proteins of coagulation [12,13], non-transfusion-use therapeutic PRP was born in the maxillofacial surgery field [14,15] as an autologous, minimally invasive approach to rapidly expand into musculoskeletal tissue, skin and ocular surface pathologies [16,17]. With the global development of PRPs, the pioneering works on autologous blood of Bob Marx [15], who used large volumes, and Eduardo Anitua [14], adopting small volumes and removing red and white blood cells, were the spark that ignited the subsequent scientific effort of research groups and technology companies in this area of regenerative medicine [14,15]. PRP consists of an autologous concentrate of platelets suspended in plasma that, once activated, becomes a fibrin biomaterial soaked up by growth factors coming from platelets and plasma, basically replicating coagulation and hemostasis but without an inflammatory response [14,17].

## 2. Immunothrombosis

Following acute injury, infection or tissue hypoxia, our body responds with an acute response phase (APR) with two consecutive multifaceted processes [18,19,20]. The first is the hemostatic–inflammatory phase, where the immunothrombosis plays a central survival role to deal with injuries and infections, resulting into the convergence of inflammation, thrombosis and hemostasis [21,22,23]. This first complex event is followed by the repair process initiated through the resolution of inflammation and multilayered, non-linear cell reprogramming, which leads to tissue restitution [18,19,20].

**Figure 1:**
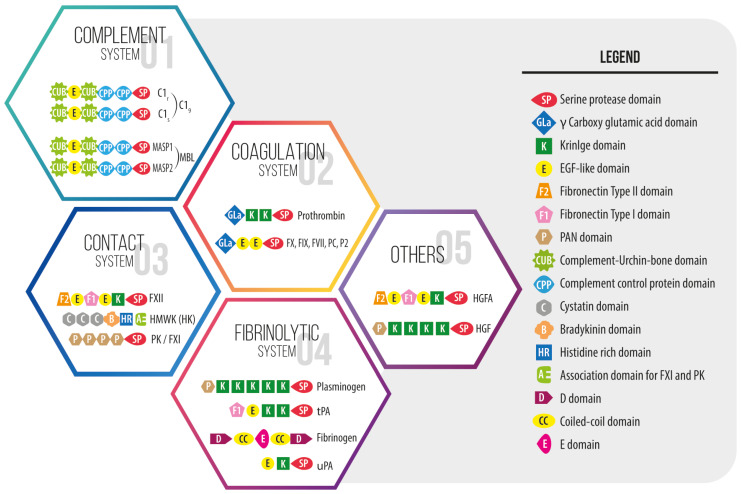
Domain organization of some multidomain proteins that make up the intravascular innate immune cascade systems, with the catalytic serine protease domain as a key enzymatic region. Reproduced from [43].

Immunothrombosis is initiated by exposure of the subendothelial tissue factor (TF) to blood due to injury or infection. It is driven by the conversion of fibrinogen to fibrin by thrombin, which occurs in the presence of Ca^2+^ ions on already aggregated and activated platelets and endothelial cells, providing a scenario for coagulation enzymatic reactions [24]. This thrombotic event is tightly linked to the activation of inflammatory pathways, including the complement and the contact systems [25,26], by FXII as one of the significant plasma protein connectors between thrombosis and inflammation [25,26,27]. Several multidomain signaling proteins of the complement, coagulation and contact systems including but not limited to thrombin, fibrin(ogen), FXII, Kallikrein, tissue factor, C3 convertase, C3a and C5a anaphylatoxins [22,25,28], play crucial roles in this first phase. These multifunctional proteins, together with platelets, initially operate locally, vigorously and rapidly, leading to thrombus generation, which compartmentalizes the damage, curtails the hemorrhage and avoids the spread of micro-organisms [22,23,25,27,29,30] (Figure 1).

The second phase of the APR is dominated by fibrinolysis, which involves the gradual clearance of the provisional fibrin meshwork of the thrombus by a series of proteases, including the plasminogen–plasmin axis and tissue metalloproteases [19,31]. This process is also driven by the pleiotropic proteins of the HGFA-HGF-MET signaling pathway, which play multifaceted roles in inflammation, coagulation and wound healing [32,33,34,35]. This pathway contributes to the bridging of the gap between survival and repair processes, paving the way for the resolution of inflammation and tissue repair. Key processes include macrophage and neutrophil polarization, fibroblast–myofibroblast differentiation and Schwann cell/repair Bugner cell transdifferentiation, among others [19,20,36,37,38]. Importantly, the fibrin matrix comprises not only the thrombus skeleton, which traps white and red blood cells, and platelets [30], sometimes wreaking havoc systemically [39,40] through pathological thrombosis and systemic inflammatory response syndrome [39,41], but it also operates as a local bridge between the hemostatic inflammatory phase, fibrinolysis and tissue repair [20,42].

## 3. PRPs: Uncoupling Inflammation and Thrombosis for Tissue Repair

**Figure 2:**
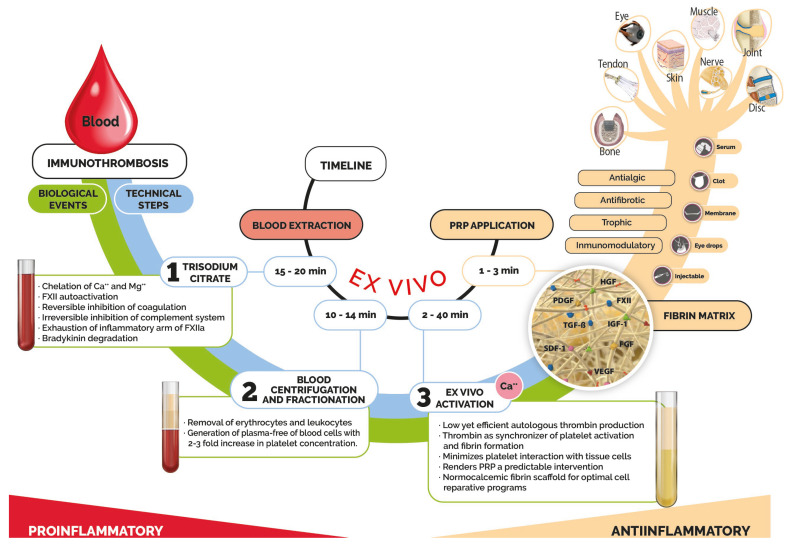
Summary of the main biological events associated with the technical steps during the processing of patient blood to manufacture platelet-rich plasma (PRP). The processing entails blood anticoagulation with sodium citrate, blood deconstruction through centrifugation and fractionation and the activation of plasma with calcium chloride. These technical steps bring about several biological events, leading to the uncoupling of inflammation and thrombosis to yield a blood-derivative formulation with anti-inflammatory, trophic, antifibrotic and antialgic effects in a context-dependent manner.

The preparation of PRP varies depending on myriad diverse commercial branches that yield heterogeneous products endowed with significantly different compositions and biological effects, all under the umbrella term PRP [44,45,46,47]. The final common formulation basically includes proteins from plasma and platelets with essential roles in tissue repair and excludes erythrocytes and leukocytes that would perpetuate inflammation and interfere with the repair process [17,29,48,49,50,51]. The underlying biological process of PRPs is FXII-driven coagulation and hemostasis as a part of immunothrombosis [17,29,52], although the mechanisms of the ex vivo uncoupling of thrombotic and inflammatory responses of PRPs remains to be fully understood. However, there are three steps in PRP manufacturing that deeply influence this dissociation [29] and, thus, contribute to anti-inflammatory, trophic, antifibrotic and antialgic effects as the hallmark of some PRPs [17,53] (Figure 2).

First, sodium citrate (SC), an anticoagulant used in the manufacturing of PRPs, chelates calcium and magnesium from plasma, thereby preventing coagulation from happening [25,54,55]. Moreover, SC disrupts the soluble proteins of the classical and lectin pathways of the complement system [25,54], inhibiting the complement system as a potent mediator of inflammation [54,56,57]. However, SC does not inhibit the activation of FXII, a calcium-independent zymogen [55,58], and FXII (FXIIa protease) is activated from the very moment that blood makes contact with the blood collection tube [52,59,60,61]. This event brings about the ex vivo activation of the inflammatory arm of the contact system and the release of bradykinin (BK) [57,61,62]. BK is a potent inflammatory mediator with a short half-life, as it is degraded in the blood collection tube itself within 30–50 s by angiotensin-converting enzymes (ACEs) and aminopeptidases [61,62] present in the plasma, thereby minimizing the BK-mediated inflammatory potential of PRPs.

Therefore, whereas FXII-dependent inflammation takes place in the tube, FXIIa does not initiate the thrombotic arm of the contact system because the coagulation cascade is calcium-dependent [55,58]. In addition, by the time we activate the PRP with calcium (next step), then apply it, enough time may have passed (40–50 min) so that a significant part of FXIIa has been proteolytically inactivated [59,60], which may contribute to the minimization of FXIIa-mediated inflammatory and profibrotic tissue effects [27,59,63,64]. Last but not least, the use of citrate as an anticoagulant does not modify the kinetics of growth factor release from the fibrin matrix [65].

The second step is blood deconstruction through a single, moderate centrifugation process, resulting in the typical three-layer segregation of citrated blood. This centrifugation process maximizes platelet production and the sedimentation of erythrocytes and leukocytes. This keeps the upper plasma layer, which contains platelets, suspended at a concentration two to three times higher than that of whole blood while keeping it free of leukocytes, known as plasma rich growth factors (PRGF) [14,66]. Nevertheless, the type of centrifugation, fractionation and activation depends on the commercial brand, deeply impinging on the final PRP composition and therapeutic effects [67,68,69]. The exclusion of erythrocytes and leukocytes significantly contributes to PRPs’ anti-inflammatory, antifibrotic, antialgic and nonimmunogenic effects [17,48,49,51,70,71,72]. The aim of the clinical translation of this fractioning and blood-cell discarding is to minimize inflammation, pain and fibrosis, which might be triggered by leukocytes and, inevitably, erythrocytes carried by some PRPs [73,74,75,76,77,78,79,80,81]. In fact, leukocytes and erythrocytes may well operate as a second insult on low-grade sterile inflammatory pathologies targeted by PRPs and endowed with inflammatory memory [82,83], thereby perpetuating non-resolving inflammation [48,49,51,84,85].

The third key step is known as PRP activation. It involves the addition of calcium chloride (CaCl_2_), which restores plasma calcium. This enables ex vivo coagulation to occur through the thrombotic arm of the contact system, given that FXII has already been autoactivated (FXIIa) upon contact with the blood collection tubes [55,58,86]. The central event is the generation of low but efficient and sufficient levels of native autologous thrombin [55,58] on platelet surfaces [87,88,89], where this serine protease catalyzes the conversion of fibrinogen to fibrin [57,90]. In doing so, thrombin operates as the synchronizer of platelet activation and fibrin formation, triggering the degranulation and release of platelet growth factors, cytokines, chemokines, adhesive proteins and microparticles [91,92,93], which, together with plasma proteins, bind transiently to the heparan sulphate proteoglycan domains (HSPGs) of fibrin [94,95,96]. Importantly, thrombin is a pleiotropic multidomain protein that, at low concentrations, exhibits growth factor-like fibroblast and endothelial cell proliferation, migration, antiapoptotic and inflammatory modulation activities [97,98,99,100]. Thrombin signaling is necessary in wound healing [97,98,99,100], where thrombin activates platelets, as well as the IL-1α and HGFA-HGF-MET signaling pathways, generating active HGF, thereby functioning as a link between injury and repair [34,99]. Simultaneously, the native thrombin is the most potent activator of platelets, facilitated by the presence of extracellular calcium [87,88,101]. Activated platelets are integrated into the fibrin matrix during its formation process, inducing its retraction and releasing the platelet payload into the fibrin matrix [91,92,102]. In this scenario, platelets exhibit a reduced reactive capacity with the cells present in the injured tissue. [30,103].

Moreover, the application of activated PRP results in the formation of a normocalcemic fibrin matrix, which is essential for the migration and proliferation of cells, paving the way for tissue reconstruction [104,105]. In addition, calcium is involved in the process of repair [106,107], as well as in cell homeostasis and signaling [108,109,110].

## 4. What Can Kill You Can Heal You—Harnessing Coagulation to Heal: Scientific Rationale Behind PRP

**Figure 3:**
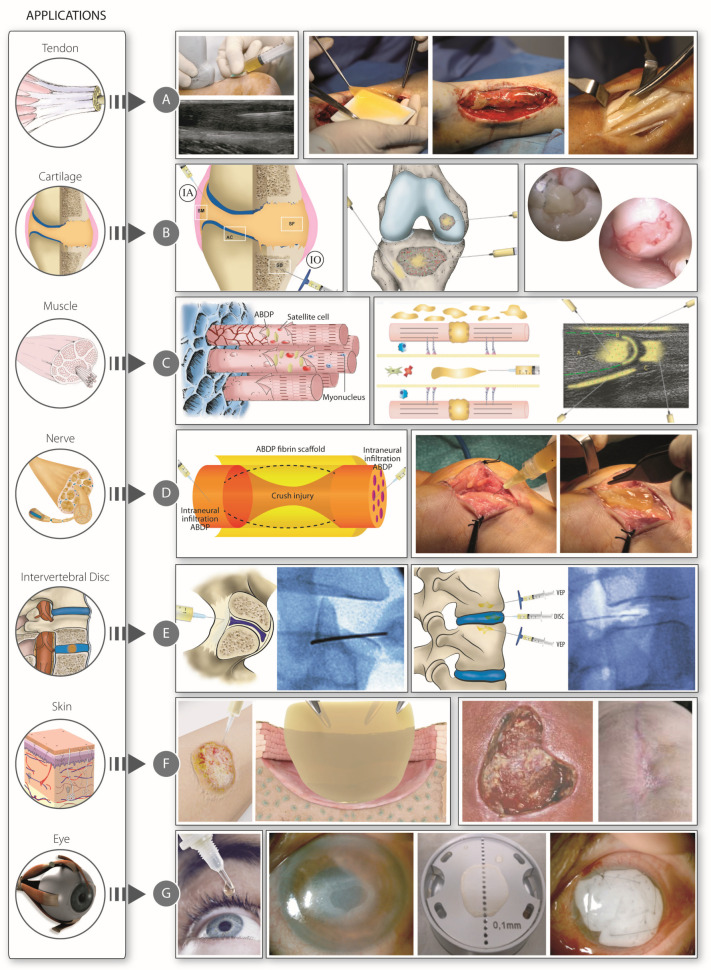
Application of PRP in different tissues and pathologies. Reproduced with permission from [17].

The process of manufacturing PRP, as previously described, not only removes the inflammatory, catabolic and immunogenic potential of some blood cells and biomolecules but can also pave the way for the myriad pleiotropic proteins to exert anti-inflammatory, trophic, reparative, antifibrotic and antialgic effects [17,53]. This biology-as-a-drug approach uses multidomain proteins of the coagulation and fibrinolytic systems, including thrombin; fibrin(ogen); plasmin(ogen); and other phylogenetically and structurally related serine proteases, such as C3 convertase, HGFA and HGF, together with platelet-derived biomolecules. Altogether, these proteins comprise a biological system with pleiotropic roles in wound healing [17,32,33,34,35,63,64,97,100,111,112]. The central components of PRPs are the growth factors and biomolecules trapped in a functionalized fibrin matrix after activation with calcium. Some of these biological mediators come from platelets (TGF-β, PDGF, VEGF, FGF, HGF, IGF-1, CTGF, BDNF and SDF-1α), and others come primarily from plasma (HGF, IGF-1, fibrin(ogen), plasmin(ogen) and α-Macroglobulin). The fibrin serves as a reservoir and carrier of these mediators, as well as a provisional three-dimensional scenario supporting surviving cells and attracting neighboring cells through fibrin-cell adhesion receptors and growth factors (VEGF and SDF-1) [102,113,114]. Generally, it is injected as a liquid-to-gel dynamic scaffold, since this phase transition is brought about when 15–20% of the fibrinogen has been incorporated into the gel by branching points, which takes around 4–5 min, known as the gel point [115]. It then takes a further 20 min to obtain the membrane and clot formulations applied in surgery (Figure 2) [17]. As a liquid, PRP extensively permeates through injured areas as a 3D fibrin-extracellular matrix-like malleable structure [17,116]. Once in the tissue, a battery of proteases including tissue plasmin initiates fibrin biodegradation, releasing a myriad of biological mediators both immediately and in a gradual and delayed manner over several days. This process operates as a biomimetic, biphasic GF delivery system [117,118,119]. In doing so, a fibrin matrix guides multi-scale molecular and cellular events to enhance tissue repair and minimize pain, inflammation and fibrosis [66,70,112,120,121]. The biological effects of complex, nonlinear, dynamic interactions between GFs and cells, as well as between GFs themselves, range from tissue repair capacity and stimulation of angiogenesis and neurogenesis to modulation of fibrogenesis and myogenesis [17,19,20]. These effects also include antialgic, antifibrotic and immunomodulatory properties [17,29,53,122] in musculoskeletal pathologies associated with sterile, low-grade inflammation. The therapeutic applications of PRP with beneficial pain reduction and functional improvement include knee osteoarthritis, tendinopathies, chronic intervertebral disc pathology, peripheral neuropathies, skin injuries, several dental conditions and ocular surface conditions [17,29]; more recently, PRPs have been used as adjuvants in assisted reproduction technology [123,124] (Figure 3). Accordingly, the manufacturing process of some PRPs, as is the case of PRGF, offers, in addition to a liquid-to-gel dynamic scaffold, other formulations that make the biologic product very versatile (Figure 2) [17]. For instance, the removal of fibrin leads to the attainment of a supernatant that can yield several other therapeutic formulations while maintaining the pool of bioactive molecules [124,125,126,127,128].

This is the case for its use as a supplement for in vitro stem-cell culture, making it a candidate to replace xenogeneic reagents such as fetal bovine serum (FBS) [129]. More importantly, this supernatant applied topically as eye drops or customized eye drops with reduced immunogenic components has been widely and successfully used to treat several ocular surface disorders, including dry eye disease, ocular ulcers or persistent ocular defects, Sjögren’s syndrome, graft-versus-host disease and cicatrizing conjunctivitis [125,128]. In addition, the supernatant formulation has been applied as adjuvant in assisted reproductive medicine, particularly in the context of infertility treatment. Accordingly, recent studies suggest that intraovarian and endometrial administration of a type of leukodepleted PRP (PRGF) may enhance ovarian function in patients exhibiting diminished ovarian reserve and improve endometrial receptivity in cases of recurrent implantation failure [123,124,126,127]. In general, PRP is not stored, as it is applied immediately after preparation. However, the supernatant of PRGF without fibrin (the exudate or supernatant that is produced once the PRGF has coagulated and retracted) can be used as eye drops or a supplement of culture media for ex vivo cell expansion.

Stability studies have shown that this formulation can be stored at −20 °C for at least one year [130]. Moreover, the potential for storage at room temperature in freeze-dried form for a period of 24 months has also been demonstrated more recently [131]. Topical PRGF serum for cutaneous applications, based on albumin denaturation, can also be stored at 4 °C for at least six months [132].

## 5. Looking Ahead: There Is Plenty of Room and Hope for Improvement in the Evolving Field of PRPs

The clinical translation of these biomimetic therapeutic products is nevertheless complex. This is because the association between PRP application and clinical benefits, such as reduced pain, inflammation and fibrosis and improved organ function in tissues like tendons, muscles and synovial joints, is multifactorial.

Moreover, PRPs challenge the conventional mechanistic and reductionist dose–response approach in medicine, as the biomolecules of these blood derivatives operate as a dynamic, nonlinear, combinatorial, synergistic and multidirectional system [17]. Therefore, any variable that could potentially modify the final biological composition of a PRP (manufacturing process, age, sex and lifestyle of patient; interindividual variability; or drugs, among others) or the interaction of PRP with damaged tissues (modalities or protocols of application; number, timing and volume of applications; and different types of pathology) would potentially result in different clinical outcomes [44,133,134,135,136]. Although PRPs are safe blood derivatives [14,29,128], there are some limitations in their use. Age, as well as several health conditions, such as immunocompromised conditions, diabetes, obesity, gout, sedentarism and unhealthy nutrition, is a factor that may profoundly impinge on diverse blood components, rendering these autologous blood derivatives inefficient [6,7,8,50,137,138,139,140,141]. Further proteomic studies are needed to unravel differences in PRP content based on factors such as age, gender, lifestyle and comorbidities and to establish links between these factors and clinical outcomes. Specifically, several associations have been found in PRGF treatment. In 2025, Sanchez et al. found an association between sex and PRGF treatment, which provided protection against the development of arthrofibrosis following anterior cruciate ligament surgery [142]. Delgado et al. [143] found that younger PRPs had more powerful anti-inflammatory and anti-apoptotic effects due to their higher concentrations of anabolic growth factors than older PRPs. However, Anitua et al. [144] found no differences in growth factors or antifibrotic response against sun-derived photo-oxidative stress between young and middle-aged individuals. More recently, Sánchez-Santiuste et al. [145] found a positive correlation between the response to intraosseous treatment with PRGF for knee osteoarthritis and age. Regarding nutritional status, De Luca et al. published a comprehensive article in 2025 examining the impact of nutritional status on the clinical outcomes of PRGF treatment, emphasizing the effects of obesity and metabolic disorders on PRGF composition [141]. Overall, more comprehensive studies are needed to systematically examine the relationships between PRGF composition, patient demographics and health variables, and clinical outcomes.

The substantial gap between the potential clinical benefits of PRPs and their specific medical indications demands significant effort among medical specialists, researchers and industry. One way to reduce this gap and circumvent some of the aforementioned pitfalls derived from patients’ conditions is the use of allogeneic PRPs by generating a pool of PRPs from specific young, active and healthy donors. Since the immunogenicity of blood resides primarily in the protein of blood cell membranes [85,146,147,148,149], the process of removing red and white cells may contribute to reductions in the antigenicity of blood, thereby minimizing risk and rendering the use of allogeneic PRPs a safe therapeutic strategy [85,146,147,148,149]. Another concern to be addressed in the field of PRPs is the inconsistency of clinical results derived primarily from studies that compare dissimilar PRPs and modalities of applications [137,150,151,152,153,154,155]. These pitfalls might partially be solved by characterizing the biological composition of PRPs, accurately describing both the modalities of application in research and clinical studies and the degree of pathology [137,152] so researchers can compare studies with similar PRPs and protocols [137,152]. In this direction, several attempts have been made to standardize manufacturing processes, the protocols and modalities of their applications, and medical indications and contraindications in a form of guidelines for specific types and degrees of pathologies (osteoarthritis, tendinopathies, muscle tears, etc.) [152,156,157]. Accordingly, due to the nature of PRP therapy, which operate as biology-as-a-drug approach in a biological system far from conventional drugs, it is difficult to gather clinical evidence by only using the gold standard of evidence-based medicine: randomized clinical trials and systematic reviews and meta-analyses. In this respect, other types of studies, such as real-world evidence studies, have been proposed for the generation of more valuable evidence [136,158]. Another important issue is that patients who are going to be treated with autologous PRPs should receive recommendations about the type of diet and exercise to practice 24–48 h before donation [133]. Last but not least, it should be remembered that, as with any medical procedure, adverse events may occur. Sometimes, these may be related to the PRP administration procedure itself, while at other times, they may be due to issues with how the PRP is handled during the manufacturing process [159]. To reduce the risk of adverse effects, it is important to strictly adhere to the PRP collection procedures established by the kit manufacturer and implement pharmacovigilance protocols.

## 6. Conclusions

In summary, blood is an endless goldmine from which to extract both knowledge about how proteins and other signaling molecules guide cell behavior and raw material with which therapeutic products for tissue repair and regeneration can be manufactured. In this way, PRPs are emerging as a safe and effective versatile network of multidomain proteins with immunomodulatory, trophic, antifibrotic and analgesic effects. As described in this work, each of the technical steps, from anticoagulation and blood centrifugation and fractionation to plasma activation, entails profound physiological modifications that deeply impinge on the therapeutic outcomes associated with the final biological composition. Tailoring PRP composition and the modality of application to each type of pathology and yielding allogenic PRPs to overcome patient conditions are among the coming challenges in this promising field of blood-derivative biomimetic products.

## Data Availability

Data is unavailable due to privacy or ethical restrictions.
